# Comment on “Original Solution for Middle Ear Implant and Anesthetic/Surgical Management in a Child with Severe Craniofacial Dysmorphism”

**DOI:** 10.1155/2016/2859051

**Published:** 2016-07-25

**Authors:** Ivo Joachim Kruyt, Ann-Louise Mc Dermott, Myrthe Karianne Sophie Hol

**Affiliations:** ^1^Department of Otorhinolaryngology, Radboudumc, 6500 HB Nijmegen, Netherlands; ^2^Department of Otorhinolaryngology, Birmingham Children's Hospital, Birmingham B4 6NH, UK

With great interest we read the recently published article by Bianchin et al. describing a case report of a child with Van Maldergem Syndrome causing severe craniofacial dysmorphism and bilateral congenital conductive hearing loss due to microtia and external auditory canal atresia [1]. Auditory restoration with a conventional skin-drive bone-conduction device, attached to a steel spring headband, was not optimal causing a speech development delay. Therefore, it was decided to implant Vibrant Soundbridge. The workup to this decision and postoperative outcomes are being discussed. We agree to the statement of the author that the best solution should be provided after careful multidisciplinary assessment about risk and benefits of all possible treatments. But with this letter we would like to express our concerns regarding the incomplete considerations about the BAHA, further described as bone implant (BI), and Vibrant Soundbridge made in the article especially in such a young child with complex needs.

In the case report, clinical evaluation at five years of age showed a speech development delay, despite the application of traditional bone-conduction hearing aids from the age of twenty-two months. During preoperative pure-tone audiometry, good functional gain with hearing aids was detected, but good compliance was lacking and the device was not used correctly. This can be explained by the fact that steel spring headband was being used. Zarowski et al. stated that this type of head band should not be used for more than 1-2 hours, because of unpleasant pressure and pain caused by the tight fitting [2]. To solve this problem elastic softband was introduced in 2003, exerting significantly less pressure on the skull, increasing comfort, without affecting audiological results [3–5]. This softband is currently the treatment of choice and advised to be used from 3 months of age, especially in bilateral cases. This can be used until the age of 4 years. This article describes a minimum age of implantation of 5 years, which applies to the VSB. The BAHA consensus statement from 2005 recommends pediatric implantation after the age of 3 years [6].

Without proper comfort during testing, the conventional skin-drive bone-conduction device is not representative for a percutaneous BI. Therefore, using steel spring headband for prolonged period, that is, longer than a few days or maximum 2-3 weeks of preoperative testing phase requires, is considered obsolete.

Skin dampening must also be considered during patient counseling. Differences in audiometric threshold and speech reception thresholds (SRT) found between preoperative testing with both the softband or steel spring headband and the final postoperative results are significant at high frequencies [7]. These are extremely important in speech understanding. At frequencies of 1–4 kHz, differences of 5–20 dB were found, in favour of the percutaneous BI. This reflects in an improvement in SRT of approximately 4–7 dB, translating to approximately 20–40% difference in speech discrimination scores [2]. Zarowski et al. suggested that, during a preoperative speech audiometry test, a correction factor of 4–7 dB must be applied in order to obtain a more realistic prediction of the outcome of a percutaneous BI.

An important issue, lacking in the manuscript, to consider when choosing between a bone implant and VSB is MRI safety. MRI is being used more frequently as diagnostic tool. Between 1996 and 2010 the use of MRI has quadrupled [8]. In 2014, 109.4 MRIs were done per 1000 population in USA [9]. Therefore it is highly likely that a patient of 5 years of age especially a patient with known central nervous system abnormalities as well as other comorbidities, such as this child, will need to have an MRI during his/her lifetime. The bone implant is, after removal of the external sound processor, MRI-conditional for 3 Tesla, with an image artifact of less than 1 cm [10, 11]. In contrast the VSB used in the patient is MRI-unsafe in all conditions and needs to be explanted before an MRI can be made, hence another operation under general anesthesia [12]. Even if the new VSB (VORP-503) would have been used, which is MRI-conditional for 1.5 Tesla, the image artifact is 14 cm; therefore diagnostic imaging of more than half of the head and neck is not possible [12].

Another key issue in which the VSB differs from BI is duration of surgery and therefore airway management. In both surgeries, general anesthesia is traditionally used. It must be noted that BI implantation in children younger than 10 years is generally provided as a 2-stage procedure [12]. Although this may necessitate two general anesthetics, the surgery is on average 20–30 minutes per stage and so laryngeal mask airway (LMA) can be used. VSB implantation by comparison takes considerably longer time (up to 2 hours) and so a conventional endotracheal tube is necessary for safe anesthesia. The child in this report had required previous tracheostomy and orthognathic surgery so the airway was a significant risk for endotracheal intubation. In cases where children have craniofacial abnormalities associated with a grade 3 or 4 airway (Cormack and Lehane classification) the LMA is associated with fewer perioperative airway complications, in comparison to the conventional endotracheal tubes in pediatric airway management [13–15].

Further issues are the inherent risks of the surgical procedure. BI implantation carries significantly less surgical risk with low morbidity when compared to VSB implantation and it is essentially a “reversible” procedure. The percutaneous abutment can simply be removed when the child is older and so will not compromise future implant choices. This is an extremely important discussion to have with the carer and parents of any child with the hearing loss and a complex medical condition described as in this article.

We believe that in this time of rapidly developing technology in the field of implantation otology, it is even more essential to make a shared and informed decision with the patient and caretakers. The long term implications of all the possible hearing solutions offered must be considered within the multidisciplinary assessment and discussed along with the risk and benefits of treatments with the parents.

In our tertiary referral centres we have gained a large amount of expertise in order to establish the benefits of bilateral application of bone-conduction devices. The current generation of bone implants shows improved skin tolerance and good survival rates in children [16]. It commences with softband and then bilateral simultaneous implantation at age of 4 years.

Finally, we would question whether all risks and benefits were discussed in this reported case. Furthermore this decision was made with suboptimal presurgical audiological assessment. We were very concerned that such a young child with complex medical conditions was implanted with a VSB despite the higher anesthetic/surgical risks, huge implications for future imaging, implications for future craniofacial surgery/microtia surgery, and of course the inevitable compromise this will now have on future hearing choices.

## References

[1] Bianchin G., Tribi L., Reverzani A., Formigoni P., Polizzi V. (2015). Original solution for middle ear implant and anesthetic/surgical management in a child with severe craniofacial dysmorphism. *Case Reports in Otolaryngology*.

[2] Zarowski A. J., Verstraeten N., Somers T., Riff D., Offeciers E. F. (2011). Headbands, testbands and softbands in preoperative testing and application of bone-anchored devices in adults and children. *Advances in Oto-Rhino-Laryngology*.

[3] Verhagen C. V. M., Hol M. K. S., Coppens-Schellekens W., Snik A. F. M., Cremers C. W. R. J. (2008). The Baha Softband. A new treatment for young children with bilateral congenital aural atresia. *International Journal of Pediatric Otorhinolaryngology*.

[4] Hodgetts W. E., Scollie S. D., Swain R. (2006). Effects of applied contact force and volume control setting on output force levels of the BAHA® Softband. *International Journal of Audiology*.

[5] Hol M. K. S., Cremers C. W. R. J., Coppens-Schellekens W., Snik A. F. M. (2005). The BAHA Softband: a new treatment for young children with bilateral congenital aural atresia. *International Journal of Pediatric Otorhinolaryngology*.

[6] Snik A. F., Mylanus E. A., Proops D. W. (2005). Consensus statements on the BAHA system: where do we stand at present?. *Annals of Otology, Rhinology & Laryngology, Supplement*.

[7] Christensen L., Smith-Olinde L., Kimberlain J., Richter G. T., Dornhoffer J. L. (2010). Comparison of traditional bone-conduction hearing aids with the Baha® system. *Journal of the American Academy of Audiology*.

[8] Smith-Bindman R., Miglioretti D. L., Johnson E. (2012). Use of diagnostic imaging studies and associated radiation exposure for patients enrolled in large integrated health care systems, 1996–2010. *The Journal of the American Medical Association*.

[9] http://stats.oecd.org/.

[11] Fritsch M. H., Naumann I. C., Mosier K. M. (2008). BAHA devices and magnetic resonance imaging scanners. *Otology and Neurotology*.

[12] MED-EL Safety recommendations and guidelines for MRI scanning. http://www.medel.com/isi.

[13] Chen Y.-L., Wu K.-H. (2009). Airway management of patients with craniofacial abnormalities: 10-year experience at a teaching hospital in Taiwan. *Journal of the Chinese Medical Association*.

[14] Banga R., Reid A. P., Proops D. W., McDermott A., Stokes M. A. (2014). Perioperative considerations for children undergoing bone anchored hearing device surgery: an observational study. *European Archives of Oto-Rhino-Laryngology*.

[15] Jones S. E. F., Dickson U., Moriarty A. (2001). Anaesthesia for insertion of bone-anchored hearing aids in children: a 7-year audit. *Anaesthesia*.

[16] Doshi J., Sheehan P., McDermott A. L. (2012). Bone anchored hearing aids in children: an update. *International Journal of Pediatric Otorhinolaryngology*.

